# Advances in the study of mechanisms underlying olfactory dysfunction in obstructive sleep apnea:a narrative review

**DOI:** 10.1007/s41105-025-00582-z

**Published:** 2025-04-03

**Authors:** Qihang Zhang, Guangling Zhang, Pengfei Ba, Tianyi Wu, Guangke Wang

**Affiliations:** 1 Department of Otorhinolaryngology and Head and Neck Surgery, Zhengzhou University People’s Hospital, Zhengzhou, China; 2 Department of Otorhinolaryngology and Head and Neck Surgery, Henan Provincial People’s Hospital, Zhengzhou, 450000 China; 3 Department of Otorhinolaryngology and Head and Neck Surgery, Henan Provincial People’s Hospital, Zhengzhou, China

**Keywords:** Obstructive sleep apnea, Olfaction disorders, Quality of life, Olfactory function

## Abstract

Obstructive sleep apnea (OSA) and olfactory dysfunction have a reciprocal relationship, where each condition exacerbates the other. This review aims to systematically explore the key mechanisms underlying the association between OSA and olfactory impairment, offering a detailed analysis of these interrelated phenomena. A comprehensive literature search was conducted on PubMed/MEDLINE in October 2024, focusing on studies published in the last decade that investigate the link between OSA and olfactory dysfunction. The search included various types of research, such as review articles, experimental studies, and clinical trials, which examine the interplay between OSA, sleep disturbances, intermittent hypoxia, and olfactory dysfunction. Literature analysis indicates that oxidative stress, inflammatory responses, central nervous system damage, and nasal airflow obstruction caused by OSA are the primary causes of olfactory dysfunction. Furthermore, these mechanisms exhibit complex interactions, suggesting a multifactorial etiology in which both peripheral and central factors contribute to olfactory impairment. There is a strong association between OSA and olfactory dysfunction. However, current research predominantly focuses on basic experimental findings, with a noticeable lack of robust clinical trial evidence. Future research should prioritize clinical investigations to further validate these mechanisms and guide the development of targeted therapeutic interventions.

## Introduction

Obstructive sleep apnea (OSA), also referred to as obstructive sleep apnea–hypopnea syndrome (OSAHS), is a prevalent pathological condition occurring during sleep, characterized by repeated episodes of apnea and hypopnea. The clinical manifestations of OSA include loud, irregular snoring, episodes of gasping for air or sudden awakenings during sleep, and daytime symptoms such as excessive drowsiness, memory impairment, cognitive confusion, and, in severe cases, cognitive decline. OSA is widely recognized as a systemic disorder and an independent risk factor for a range of conditions, including coronary artery atherosclerosis [1].Olfactory dysfunction, on the other hand, encompasses abnormalities in olfactory perception due to damage at various stages of the olfactory pathway. These may include functional impairments in the olfactory receptor neurons, damage to the olfactory bulb, or structural and functional alterations in the olfactory cortex [2].

### Correlation between olfactory dysfunction and obstructive sleep apnea

In recent years, with the continuous development of the economy and improvements in living standards, the global prevalence of obstructive sleep apnea (OSA) has shown a marked upward trend. The associated complications, such as impaired traffic safety, sudden death, and reduced productivity, have increasingly become major public health and social concerns [1]. Moreover, a growing body of evidence indicates that patients with OSA often experience varying degrees of olfactory dysfunction. This review aims to explore the mechanisms contributing to olfactory dysfunction in OSA patients, with the goal of providing a foundation for targeted treatments and offering clinical guidance for managing OSA patients who present with olfactory impairment.

## Mechanisms of olfactory dysfunction in obstructive sleep apnea

1.Oxidative stress in OSA patients leads to olfactory dysfunction.

Reactive oxygen species (ROS) refer to substances with oxidative activity generated within cells. The primary pathways for their formation involve the reduction of a portion of O2 to O2- or H2O2 during the electron transport process in the inner mitochondrial membrane, or the generation of ROS through the electron transport process catalyzed by the NADPH oxidase subunit (NADPH oxidase 2, NOX2/gp91phox) on the cytoplasmic membrane. Reactive nitrogen species (RNS), on the other hand, are nitrogen-containing oxidants such as NO and HNO3 produced by mitochondria under hypoxic conditions. "Oxidative stress" refers to the dynamic imbalance between the antioxidant and oxidative systems within the organism, resulting in the excessive accumulation of free radicals like ROS and RNS. These free radicals can damage lipids, proteins, DNA, and carbohydrates within the body, thereby causing molecular-level damage to the organism's functions.

Alzheimer's disease (AD) is a degenerative disorder of the central nervous system characterized by progressive cognitive dysfunction and behavioral impairment, which may be closely associated with olfactory dysfunction. Researchers such as Zuo [3] conducted a cross-sectional study on the levels of oxygen free radicals in the serum of Parkinson's patients with olfactory dysfunction. They found that patients with olfactory dysfunction had higher concentrations of hydroxyl radicals (•OH) and lower concentrations of H2O2 in their serum compared to those without olfactory dysfunction. Zuo hypothesized that this phenomenon might result from the conversion of more H2O2 into hydroxyl radicals, leading to damage to olfactory neurons.Laura et al. [4] evaluated the potential effects of vanadium, a toxic substance capable of inducing oxidative stress, on the olfactory function of mice. The experiment involved exposing mice to 0.02 M vanadium pentoxide via inhalation for four weeks. The mice were euthanized at 1, 2, 3, and 4 weeks post-exposure, while the control group inhaled physiological saline. The study aimed to assess the impact of vanadium-mediated oxidative stress on olfactory function and related enzyme activities. The results indicated a statistically significant difference in olfactory function between the control and exposed groups. Compared to the control group, the exposed group showed a significant increase in odor localization time after four weeks of vanadium pentoxide inhalation. Microscopic observations revealed a loss of dendritic spines in the granular cells of the olfactory bulb across all exposure periods, with a sharp decline in dendritic spine density by the fourth week. Additionally, neuronal lipofuscin deposition, Golgi apparatus vacuolization, and concurrent apoptosis and necrosis were observed in the mice. Analysis of the olfactory bulb's antioxidant components, glutathione reductase (GR) and glutathione peroxidase (GPx), showed that GPx activity significantly increased within two weeks compared to the control group but declined after four weeks, though not statistically significant. In contrast, GR activity significantly increased at both two and four weeks compared to the control group. These findings suggest that vanadium exposure may disrupt the olfactory system by inducing oxidative stress, damaging neuronal structures, and depleting intracellular reductases.

Due to the characteristics of OSA patients, such as obesity or abnormal anatomical structures, the cross-sectional area of the upper airway may be reduced in OSA patients, leading to incomplete pulmonary gas exchange during sleep and causing hypoxemia and hypercapnia [1]. Research has revealed that oxidative stress is prevalent among OSA patients, primarily because the rapid changes in oxygen tension in these patients are considered analogous to hypoxia-reoxygenation injury [6]. This alternating process of hypoxia and reoxygenation can affect the composition of body cells, promoting the production of reactive oxygen species (ROS) and damaging various bodily functions, including olfaction. For instance, ROS can affect nucleic acid stability by oxidatively damaging DNA/RNA nucleotide heterocyclic bases; disrupt protein structures through carbonylation and nitration [7]; and alter the release of dopaminergic neurons by abnormally activating ATP-sensitive potassium channels and inactivating acetylcholine receptors [8]. In addition, oxidative stress can directly or indirectly affect intracellular components such as proteins and lipids through various intracellular signaling pathways, including NF-κB, NRF2, JAK-STAT, JNK, FOXO, and HIF [5]. This process can disrupt cellular structures and even induce apoptosis [9]. Therefore, oxidative stress in patients with obstructive sleep apnea (OSA) may be associated with the occurrence of olfactory dysfunction.

2.Inflammatory response in osa patients leading to olfactory dysfunction

OSA not only induces oxidative stress in the body but also disrupts olfactory function by activating numerous inflammatory pathways. Existing literature indicates that OSA patients can trigger the activation of hypoxia-inducible factors (HIFs) due to intermittent hypoxia, which in turn activates downstream signaling pathways such as NF-kB [10–12]. This activation promotes the production of inflammatory cytokines including IL-1β, IL-2, IL-6, IL-8, TNF-α, and VEGF, which are involved in inflammatory responses and mediate neuronal death [13, 14].

(I) The Impact of Interferon and Tumor Necrosis Factor-like Inflammatory Factors on Olfaction.

Jang and Pak [15] conducted a clinical cross-sectional study on the levels of inflammatory factors in the olfactory cleft (OC) mucus samples of patients following SARS-CoV-2 infection. They collected 35 clinical samples, including 20 patients with persistent olfactory dysfunction lasting more than 3 months after SARS-CoV-2 infection, 8 patients without olfactory dysfunction after SARS-CoV-2 infection, and 7 healthy controls who had never been infected with SARS-CoV-2. The study measured 13 viral and inflammatory biomarkers in the OC mucus samples. They found significant differences in the expression of TNF-α (p = 0.049), IFN-λ1 (p = 0.007), IFN-λ2/3 (p = 0.012), and IFN-γ (p = 0.006) among the three cohorts. The cohort with persistent olfactory dysfunction after SARS-CoV-2 infection exhibited the highest concentrations of inflammatory factors. Further analysis of individual inflammatory factors revealed that the levels of IFN-α2 and IFN-γ were higher in the cohort with persistent olfactory dysfunction compared to the cohort without olfactory dysfunction after SARS-CoV-2 infection. After adjusting for multiplicity using the Benjamini-Hochberg (BH) procedure, significant differences in the expression levels of IFN-λ1 and IFN-γ were observed among the groups. However, no differences were found in the expression levels of IL-6 in the OC mucus among the three cohorts. Additionally, no viral RNA was detected in any of the cohorts, suggesting that local inflammatory responses may persist even after viral clearance, and that interferon pathway-related inflammatory factors play a critical role in SARS-CoV-2-related persistent olfactory dysfunction, Hao et al. [16] selected TAK-242, an inhibitor of Toll-like receptors (TLRs), which play a crucial role in immune and inflammatory responses, to treat olfactory dysfunction in mice with allergic rhinitis. Their study established an allergic rhinitis mouse model using ovalbumin (OVA) and employed the buried food pellet test (BFPT) to assess olfactory function. Mice exhibiting olfactory dysfunction were randomly divided into a TAK-242 treatment group and a control group. The treatment group received intraperitoneal injections of TAK-242 solvent, while the control group was injected with 1% DMSO solvent. RT-PCR analysis revealed a significant decrease in TNF-α mRNA expression in the olfactory bulb tissue of the treated mice. Additionally, microscopic observation showed a reduction in olfactory bulb cell apoptosis, accompanied by a noticeable improvement in olfactory function. Sousa and colleagues, by knocking out the tumor necrosis factor receptor TNFR1 in mice, observed in subsequent TNF-α induction experiments that the olfactory epithelium did not exhibit inflammatory responses, and no abnormal feedback was detected in the electro-olfactogram (EOG) [17]. Based on this finding, they proposed a hypothesis that TNF-α-induced olfactory neuron dysfunction may occur through a TNFR1-dependent mechanism. Considering the widespread expression of tumor necrosis factor in the cerebral cortex and hippocampus, and its ability to activate the NF-kB pathway, leading to a downstream cytokine storm and cytotoxic responses, ultimately resulting in neuronal apoptosis in the cerebral cortex and hippocampus [18, 19].

(II) The Impact of Interleukin Inflammatory Factors on Olfaction.

In addition, factors such as IL-6 can regulate the release of more IL-6 and related downstream inflammatory factors, such as IL-1β, TNF-α, and IL-8, through the IL-6/JAK/STAT3 signaling pathway, thereby triggering a "cytokine storm" that contributes to neuronal toxicity [20, 21]. Furthermore, researchers such as Song and Sun have found that the levels of IL-6 in the olfactory bulbs of mice with olfactory dysfunction are abnormally elevated and positively correlated with the severity of the dysfunction. In vitro experiments have shown that the expression of the neuroinflammatory marker IL-1β in the supernatant of microglial cells cultured with IL-6-conditioned medium significantly increases. This finding further confirms the close association between IL-6 and olfactory dysfunction [22]. A clinical trial study conducted by Soler[23] investigated the relationship between olfactory cleft (OC) mucus inflammatory proteins and olfaction in chronic rhinosinusitis (CRS). The study collected OC mucus samples from 62 patients and utilized proteomic techniques, such as enzyme-linked immunosorbent assay (ELISA), to measure the concentrations of 26 related proteins in the mucus. Concurrently, the olfactory function of the patients was assessed using the Sniffin’ Sticks olfactory test. The patients were further divided into subgroups based on the presence or absence of nasal polyps. The results revealed that 10 OC mucus proteins were associated with olfactory threshold, discrimination, and identification (TDI) scores. Further subgroup analysis indicated that in the chronic rhinosinusitis with nasal polyps (CRSwNP) group, 9 OC mucus proteins showed correlations with olfactory TDI scores. Specifically, IL-5, IL-6, IL-13, IL-10, IL-9, TNF-α, CCL5, and CCL-11 were significantly associated with olfaction, and each of these proteins exhibited a negative correlation with olfactory function. However, in the chronic rhinosinusitis without nasal polyps (CRSsNP) group, only CXCL5 was found to be related to olfaction.This indicates that inflammatory factors such as CCL-2 and IL-5 are significantly correlated with olfactory function. Further subgroup analyses of CRSwNP and CRSsNP have confirmed the specific role of interleukin-related inflammatory factors in olfactory dysfunction. Given that CCL-2 and IL-5 are significantly negatively correlated with olfactory function, it suggests that these inflammatory factors play a detrimental role in olfactory function. This may be due to the impact of related inflammatory responses on the olfactory conduction pathway or the function of relevant receptors, thereby contributing to the decline in olfactory function. However, the specific mechanisms by which these inflammatory factors exert their effects in the CRSwNP subgroup still require further investigation and exploration. In summary, the release of interleukin-related cytokines induced by OSA can lead to the development of olfactory dysfunction symptoms.

Comprehensive analysis indicates that inflammatory factors play a significant role in the damage of olfactory neurons, possibly due to their ability to significantly alter the activity of neuronal ion channels, particularly the K + ion channels [24]. Further research reveals that inflammatory factors can also exert cytotoxic effects by inhibiting axonal growth and development, interfering with neurotransmitter release, and regulating neuroendocrine functions [25]. In summary, patients with OSA may experience recurrent intermittent hypoxia, triggering inflammatory responses, activating multiple inflammatory signaling pathways, and resulting in the release of a large number of inflammatory factors. This process ultimately may cause damage to olfactory-related neurons and their functions.

3. Neurological disorders in OSA patients leading to olfactory dysfunction

Since human wakefulness and sleep are regulated by the interaction of multiple neural systems, with the states of wakefulness and sleep primarily maintained by two major systems: monoaminergic/cholinergic (wake-promoting) neurons and γ-aminobutyric acid (sleep-promoting) neurons [26], patients with obstructive sleep apnea (OSA) experience recurrent episodes of sleep apnea and hypopnea during nighttime sleep. These episodes can enhance sympathetic nervous system activity and are accompanied by repeated arousals and arousal responses in the cerebral cortex, disrupting the normal neural regulatory rhythm [1]. The olfactory nerve, as a bipolar neuron, has dendrites that are widely distributed in the nasal mucosa to sense odor signals, while its axons form the olfactory nerve, which passes through the cribriform plate of the ethmoid bone into the anterior cranial fossa, terminating in the olfactory bulb. The olfactory nerve then projects via the olfactory tract to regions such as the uncus of the temporal lobe, the presubiculum, and the amygdala. Several studies have shown that OSA, particularly the intermittent hypoxia it induces at night, can cause varying degrees of changes and damage to the olfactory system from the periphery to the central nervous system [27–29].

(I) The Impact of Cholinergic System Dysfunction on Olfaction.

In the olfactory processing system, numerous types of neurons collaborate in the processing of olfactory signals, including the endocannabinoid system, dopaminergic system, cholinergic system, noradrenergic system, and serotonergic system [30]. Cholinergic fibers, originating from the basal forebrain, significantly modulate the plasticity of odor signals. Nicotinic and muscarinic receptor subtypes, as part of the cholinergic fibers, can regulate the inhibitory pathways of the periglomerular cells in the olfactory bulb, thereby playing a role in the perception modification and fine-tuning of olfactory signals. Additionally, muscarinic receptor neurons may also be involved in the connections between mitral cells (involved in the processing and encoding of olfactory information) and granule cells (regulating the output of the olfactory bulb to the cerebral cortex) in the olfactory bulb, which is crucial in neural activities that prioritize attention and information processing through olfaction. Studies have shown that cholinergic neurons also play a broad role in odor short-term memory, recognition of similar odors, signal relay in the olfactory bulb, and modulation [30, 31].Qian and Rawashdeh induced sleep-disordered breathing in a mouse model of familial Alzheimer's disease (AD) and found that the exacerbation of AD symptoms was accompanied by selective degeneration of cholinergic pre-basal forebrain neurons. The study also revealed that the damage to cholinergic neurons was closely associated with the accumulation of hypoxia-inducible factor 1α (HIF-1α) within cells [32], suggesting that hypoxia caused by nocturnal sleep disturbances poses a significant threat to cholinergic neurons in patients with obstructive sleep apnea (OSA). Versace and Langthaler et al. used polysomnography (PSG) to screen 20 OSA patients and 20 healthy controls matched for age and gender. They employed a paired-pulse transcranial magnetic stimulation (TMS) method known as short-latency afferent inhibition (SAI). The researchers applied a paired-pulse stimulation paradigm, primarily targeting the motor cortex. A conditioning stimulus (CS), which was subthreshold, was delivered first, followed by a test stimulus (TS), which was suprathreshold, after a certain interval. By measuring the inhibition of motor-evoked potentials at different interstimulus intervals, they assessed central cholinergic function between the two groups. Additionally, they used the Sniffin’ Sticks olfactory test to evaluate odor identification and discrimination abilities in both groups.The research results indicate that, compared to the control group, the experimental group showed a significant reduction in SAI. Additionally, OSA patients exhibited notably lower odor identification and discrimination abilities than the healthy control group, indicating olfactory dysfunction. Correlation analysis between SAI values and olfactory function tests revealed a strong negative correlation between SAI values and various parameters of olfactory function. Consequently, the researchers inferred that olfactory dysfunction in OSA patients may be closely related to dysfunction of cholinergic neurons in the central nervous system. Versace suggested that this could be due to factors such as recurrent intermittent hypoxia and sleep structure disruption caused by OSA, which affect the normal function of the central cholinergic nervous system, leading to abnormal activity of cholinergic neurons in the olfactory pathway and resulting in olfactory dysfunction. This study also introduced a novel method for assessing cholinergic function in OSA patients by measuring SAI values using TMS technology, offering new insights for further understanding OSA [33].

(II) The Impact of γ-Aminobutyric Acid System Dysfunction on Olfaction.

Gamma-aminobutyric acid (GABA) serves as a crucial inhibitory neurotransmitter in the nervous system and similarly participates in the composition of various interneurons within the olfactory bulb [29]. Granule cells in the olfactory bulb, functioning as interneurons, are the primary GABAergic neurons that inhibit the excessive firing of mitral cells, thereby regulating the input of olfactory signals to higher centers [30]. Additionally, recent findings in the central nervous system have revealed that GABA can activate metabolic GABA receptors (GBB-1) in glial cells, inducing neuroprotective effects through trophic actions, and possibly mediating olfactory adaptation via ionotropic GABA receptors (LGC-38) in glial cells [34]. In a clinical trial conducted by Macey et al., 14 patients diagnosed with OSA at the UCLA Sleep Disorders Center and 22 healthy participants matched for age and gender were selected as the control group. Magnetic resonance spectroscopy was used to measure the levels of GABA and glutamate in the bilateral insular cortex of the 14 OSA patients. The results revealed significant differences in GABA and glutamate levels in the left and right insular regions between OSA patients and healthy controls. Specifically, GABA levels in the anterior insular cortex were significantly lower in OSA patients compared to the 22 healthy controls, while glutamate levels in the same regions were higher in OSA patients. In the left anterior insular cortex, GABA levels were 0.36 ± 0.10 in OSA patients and 0.62 ± 0.18 in healthy controls. In the right anterior insular cortex, GABA levels were 0.27 ± 0.09 in OSA patients and 0.45 ± 0.16 in healthy controls. Glutamate levels in the left anterior insular cortex were 1.61 ± 0.32 in OSA patients and 0.94 ± 0.34 in healthy controls, while in the right anterior insular cortex, glutamate levels were 1.26 ± 0.28 in OSA patients and 1.02 ± 0.28 in healthy controls.Analysis of the correlation between GABA and glutamate levels within the two groups revealed a significant negative correlation in the healthy control group, whereas no such correlation was observed in the OSA group. This suggests that OSA may lead to an imbalance of neurotransmitters in the insular cortex of patients, which plays a crucial role in various functions such as emotion, cognition, taste, and olfaction. The disruption of these neurotransmitters could potentially have adverse effects on olfactory function, among others. Additionally, the experiment found that OSA patients did not exhibit the negative correlation between GABA and glutamate levels observed in the healthy control group. This may be due to altered neurochemical regulatory mechanisms within the insular cortex of OSA patients, which could reflect the neurophysiological dysfunction in the brain under OSA conditions and may play a significant role in the cognitive and emotional impairments observed in OSA patients [35].

4. Disordered nasal airflow in OSA patients leads to olfactory dysfunction

As the primary gateway of the respiratory tract, the nasal cavity plays a crucial role in olfaction and respiration. When air enters the nasal cavity, it is influenced by the resistance of the nasal valve, resulting in two distinct flow patterns: laminar flow and turbulent flow. Laminar flow is the primary component of the airflow that comes into contact with the nasal mucosa, while turbulent flow, which forms downstream of the nasal valve, consists of irregular, spiral-shaped airflow and constitutes only a small portion of the inhaled air. The significance of turbulent flow lies in its ability to more thoroughly mix the inhaled air. During sleep in patients with OSA, recurrent episodes of apnea and hypopnea are significantly influenced by abnormalities in the anatomical structure of the upper airway. Conditions such as deviated nasal septum, hypertrophic nasal turbinates, nasal polyps, and nasal tumors can lead to nasal obstruction, altering the airflow dynamics within the nasal cavity [36, 40], thereby affecting the capture of olfactory signals by olfactory receptors. Fu, Pinto, et al. [37] utilized acoustic rhinometry to measure the minimal cross-sectional area (MCA) and nasal volume (NV) in 76 adults diagnosed with obstructive sleep apnea (OSA) via polysomnography. Additionally, each participant underwent the Sniffin' Sticks olfactory test to assess olfactory threshold (OT), olfactory discrimination (OD), olfactory identification (OI), and the composite Sniffin' Sticks score (SS). The results indicated a significant correlation between the minimal cross-sectional area (MCA) and the composite Sniffin' Sticks score (r = 0.434, p < 0.001), as well as with olfactory threshold (OT) (r = 0.385, p = 0.001) and olfactory discrimination (OD) (r = 0.370, p = 0.001). However, no significant correlation was found between MCA and olfactory identification (OI) (p > 0.05). Nasal volume (NV) was also correlated with the composite Sniffin' Sticks score (r = 0.350, p = 0.002). These findings suggest that nasal structure, particularly the minimal cross-sectional area, may be one of the contributing factors to olfactory dysfunction.Human olfactory receptors primarily rely on the olfactory mucosa and olfactory cells in the nasal cavity, which are widely distributed in the olfactory cleft region maintained by the superior nasal concha and the upper part of the nasal septum. Wu, Wang, et al. [38] reconstructed a three-dimensional spatial model of the nasal cavity using CT scan data from 32 volunteers, tested the olfactory function of patients using the Sniffin' Sticks method, and employed computational fluid dynamics to investigate the correlation between various airflow parameters in the olfactory cleft and olfactory function. The results revealed that the average flow rate, average velocity, and air flow ratio were highly positively correlated with olfactory function, while the average pressure showed no correlation. Detailed analysis indicated that the strongest correlation existed between the gas flow through the olfactory cleft and olfactory function. The average velocity in the anterior olfactory cleft region showed a relatively weaker correlation with olfactory function, while the airflow velocity in the posterior olfactory cleft region gradually increased, suggesting that the airflow velocity in the posterior olfactory cleft region may have a greater impact on olfactory function. The air flow ratio was also highly positively correlated with olfactory function, with the optimal ratio observed at the initial position of the superior nasal concha. This may imply that the airflow distribution ratio in the anterior part of the middle nasal concha is crucial for olfactory function, highlighting the importance of airflow distribution in different regions. The airflow parameters in the posterior olfactory cleft region were more stable, suggesting that the stability of airflow in this region may be more conducive to olfactory recognition and analysis. The study underscores the importance of nasal cavity anatomy in olfactory function and suggests that olfactory dysfunction in OSA patients may be closely related to these factors.Ebihara conducted a study on a superior turbinate lateralization (STL) procedure, which involves eliminating the connection between the turbinate bone and the anterior wall of the sphenoid sinus to increase nasal airflow. Seven patients with olfactory dysfunction due to poor olfactory cleft ventilation underwent endoscopic sinus surgery (ESS) combined with STL. Follow-up surveys were conducted at 1, 3, and 6 months postoperatively. The study found that the cross-sectional area of the olfactory cleft (OC) was significantly larger at 3 months postoperatively compared to preoperatively. Additionally, the Open Essence and olfactory questionnaires showed significantly higher scores postoperatively than preoperatively. To compare the airflow in the OC region between patients who underwent STL and those who did not, three-dimensional reconstructions of the nasal cavity were performed. Post-simulation, it was observed that the OC region was wider, and the airflow in both axial and coronal sections was higher. Analysis of the average airflow in the OC region revealed a significant increase in airflow with the STL procedure compared to cases without STL (p < 0.05). These results indicate that the STL procedure helps improve OC airflow and promotes the free entry of odorant molecules by widening the olfactory airflow pathway [39]. In summary, nasal structural abnormalities such as nasal septum deviation and nasal polyps can significantly alter nasal airflow, particularly in the olfactory cleft region, where laminar flow decreases and turbulent flow increases [36, 40]. This results in a significant reduction in the volume of gas flowing through the olfactory mucosa, making it difficult for gas molecules to reach the relevant olfactory receptors, thereby causing olfactory dysfunction [41, 42].

5.The mutual interactions among various factors can lead to olfactory dysfunction in patients with OSA.

(I) The mutual influence of neuroinflammation and oxidative stress leads to olfactory dysfunction in patients with obstructive sleep apnea (OSA).

The aforementioned factors, including inflammatory responses, oxidative stress, neurological disorders, and nasal airflow disturbances in OSA patients, can lead to olfactory dysfunction. These factors can interact with each other, collectively affecting the patients' olfactory function. Among these, neuroinflammation and oxidative stress are two critical aspects in the onset and progression of neurodegenerative diseases, and they are closely interconnected in the pathogenesis. Inflammatory cells can secrete reactive substances that induce oxidative stress, and some reactive oxygen species (ROS) and reactive nitrogen species (RNS) can further promote intracellular signaling cascades, leading to increased expression of pro-inflammatory genes. Therefore, neuroinflammation and oxidative stress can mutually stimulate each other [43]. When OSA occurs, multiple inflammatory factors are released under the influence of neuroinflammation, which can induce microglia to elevate the expression levels of pro-inflammatory cytokines, further promoting the production of interleukin-1β (IL-1β), tumor necrosis factor-alpha (TNF-α), and nitric oxide (NO). This process is particularly mediated through the activation of the nuclear factor-kappa B (NF-κB) signaling pathway, which facilitates the release of downstream factors such as TNF-α and interleukins, ultimately leading to oxidative stress and synaptic failure [44]. Conversely, due to the pivotal role of the NF-κB pathway in oxidative stress and inflammatory responses, ROS generated by OSA-induced oxidative stress can also act as signaling molecules, activating multiple inflammation-related signaling pathways. Under the influence of the transcription factor NF-κB, these pathways initiate the expression of related inflammatory genes, releasing various inflammatory factors, thereby exacerbating the inflammatory response and indirectly worsening olfactory dysfunction in OSA patients [45].

(II) The Interaction Between Oxidative Stress and Neuroinflammation in OSA Patients Influences Neuronal Degeneration.

In addition, oxidative stress and inflammatory responses caused by OSA can collectively influence neuronal degeneration, leading to olfactory dysfunction. mtDNA, as a crucial genetic material encoding mitochondrial-related proteins in cellular mitochondria, plays a significant role in constructing the mitochondrial electron transport chain, oxidative phosphorylation, and ATP production. Due to the dramatic fluctuations in oxygen tension in OSA patients, a large amount of reactive oxygen species (ROS) is generated. These ROS can directly damage mtDNA within neuronal mitochondria. Given that mtDNA has a relatively short half-life, with minimal non-coding sequences between the genes it encodes, combined with continuous exposure to ROS, limited DNA repair mechanisms, and the absence of histone protection, mtDNA is highly susceptible to oxidative damage, ultimately resulting in deleterious mutations. When ROS accumulates in OSA patients, the accumulated mtDNA mutations may lead to reduced efficiency of the electron transport chain, decreased ATP production, and increased ROS generation [46]. Conversely, the increase in ROS may lead to further accumulation of mutated mtDNA, creating a positive feedback loop that amplifies both mtDNA mutations and ROS production, ultimately resulting in cell death. Studies have shown a close correlation between aging, the accumulation of mtDNA mutations, reduced mitochondrial function, and increased oxidative stress during the aging process in both humans and animals [47].

It is noteworthy that neuroinflammatory responses are also involved in this process. When mitochondria are damaged by reactive oxygen species (ROS), their contents are released and recognized by hexokinase (HK), a glycolytic enzyme located on the outer mitochondrial membrane. Subsequently, HK detaches from the outer mitochondrial membrane and releases mitochondrial RNA (mtRNA), which further activates the NLRP3 inflammasome and triggers the release of the inflammatory cytokine IL-1β [48]. The release of inflammatory cytokines can lead to mitochondrial dysfunction and the production of ROS, thereby forming a toxic feedback loop that exacerbates the loss of mitochondrial function and ultimately results in neuronal cell death [49].

Microglia, as a type of glial cell widely distributed in the brain and spinal cord, play a significant role in the occurrence of neuroinflammation and oxidative stress. When neuroinflammation occurs, microglia can be activated into the classical M1 (pro-inflammatory) and M2 (anti-inflammatory) phenotypes [50]. Morphologically altered M1 microglia exhibit phagocytic activity and release a large number of inflammatory factors, such as tumor necrosis factor (TNF-α), IL-6, IL-1, and nitric oxide (NO). The accumulation of these factors can lead to neuronal loss in patients [51]. In contrast, M2 microglia secrete anti-inflammatory cytokines, such as IL-4, IL-13, IL-10, transforming growth factor (TGF), and insulin-like growth factor-1 (IGF-1), by phagocytosing cellular debris or damaged neurons, thereby reducing inflammation and promoting neuronal repair [52]. Additionally, apart from being activated by neuroinflammation, microglia are also a major factor in oxidative stress within the central nervous system [53]. In summary, the interplay between oxidative stress and neuroinflammation can disrupt normal neuronal activity, playing a crucial role in olfactory dysfunction in patients with obstructive sleep apnea (OSA).

Obstructive sleep apnea (OSA) is associated with numerous complications, has a wide-ranging impact, and exhibits a high prevalence rate, making it a significant public health issue that profoundly affects society and the economy. Given the multiple and complex mechanisms underlying olfactory dysfunction in OSA patients, the prevention, prediction, and personalized treatment of this condition have become critical areas of focus for otolaryngologists and head and neck surgeons in the future.In this review, oxidative stress, inflammatory responses, neurological disorders, and nasal airflow disturbances are primarily identified as the main causes of olfactory dysfunction associated with OSA (Obstructive Sleep Apnea). However, in clinical practice, the underlying mechanisms may vary depending on the individual patient's physical condition. Therefore, it is necessary to develop appropriate early screening methods, prevention strategies, and personalized treatment plans based on the actual circumstances. By evaluating biomarkers such as the biological concentration of reactive oxygen species (ROS), the types and levels of inflammatory cytokines, and structural variations in the nasal cavity, we can identify OSA patients at high risk of olfactory dysfunction. Additionally, machine learning, as an effective method for understanding complex gene expression data to identify diagnostic biomarkers for OSA, can screen for individual genes that distinguish OSA from olfactory dysfunction and identify patients at high risk of both conditions [54]. In terms of prevention, early lifestyle interventions should be implemented. Relevant studies have demonstrated that antioxidant therapy can significantly improve endothelial function, reduce oxidative stress, and enhance sleep parameters in OSA patients [55]. Furthermore, melatonin, known for its significant anti-inflammatory properties, is also believed to inhibit neuroinflammation and improve synaptic plasticity [56]. Regarding treatment, a personalized approach should be adopted. Based on the treatment of the primary disease and the patient's test indicators, anti-inflammatory and antioxidant therapies can be administered. When necessary, nasal surgery may be performed to improve ventilation and eliminate anatomical abnormalities, thereby restoring olfactory function.

## Conclusion

Obstructive sleep apnea (OSA) is a major public health concern with a high prevalence, contributing to a wide range of complications and exerting significant social and economic burdens. This paper examines the mechanisms underlying olfactory impairment in OSA patients, focusing on four key aspects: oxidative stress, inflammatory responses, neurological disorders, and nasal airflow disturbances. OSA not only leads to upper airway obstruction but also causes repeated episodes of hypoxia and inflammation, disrupting internal homeostasis and affecting multiple bodily systems.The activation of the inflammatory NF-κB pathway plays a critical role in this process, mediating the release of downstream inflammatory factors while also contributing to oxidative stress and impairing neurological functions [57]. Consequently, olfactory dysfunction in OSA patients is likely the result of these multifactorial influences. However, due to poor patient compliance and limited screening methods, the cellular and molecular mechanisms have not been adequately validated in clinical settings. There is a notable lack of epidemiological data and large-scale cohort studies that explore these relationships in depth.As a result, developing targeted and precise treatments for OSA and its associated symptoms remains challenging. With the ongoing advancement of evidence-based medicine, further exploration is needed to better understand the mechanisms and treatment options for olfactory dysfunction in OSA patients.

The correlations between olfactory dysfunction and obstructive sleep apnea.

**Table:** 

	Clinical Mechanisms	References
Oxidative Stress in OSA Patients Leads to Olfactory Dysfunction	Factors such as obesity or anatomical abnormalities in patients with obstructive sleep apnea (OSA) can reduce the cross-sectional area of the upper airway, leading to incomplete gas exchange in the lungs during sleep. This results in episodes of hypoxemia and hypercapnia, which trigger the excessive accumulation of reactive oxygen species (ROS) and reactive nitrogen species (RNS). These free radicals can cause significant damage to essential biomolecules, including lipids, proteins, DNA, and carbohydrates, ultimately compromising various cellular functions	Colín BL et al.[4] Schönenberger MJ et al.[5]
Inflammation in OSA Patients Leads to Olfactory Dysfunction	(i) Interferon-like inflammatory cytokines may contribute to the direct destruction of olfactory epithelial tissue, leading to impaired olfactory function (ii)Tumor necrosis factor (TNF) receptors are widely expressed in the cerebral cortex and hippocampus. Activation of the NF-κB pathway can trigger a downstream cytokine storm, inducing cytotoxic responses that damage olfactory neurons and lead to olfactory dysfunction. Additionally, TNF can further impair olfactory function through its receptor, TNFR1 (iii) Cytokines such as IL-6 can mediate the release of additional IL-6 and downstream inflammatory factors, including IL-1β, TNF-α, and IL-8, through the IL-6/JAK/STAT3 pathway, thereby initiating an "inflammatory cytokine storm" that contributes to neurotoxic responses. In mouse models of olfactory dysfunction, IL-6 levels in the olfactory bulb are significantly elevated and positively correlate with the severity of the dysfunction. Furthermore, the involvement of IL-1β and IL-1 type I receptor-dependent signaling in the olfactory bulb has also been demonstrated	Jang SS et al.[15] Lv H et al.[16] Sousa GD et al.[17] Dong Y et al.[18] Yu Y et al.[19] Gupta KK et al.[20] Araújo B et al.[21] Song XY et al.[22] Niu H et al.[23]
Neurological disturbances in OSA patients lead to olfactory dysfunction	(i) Patients with obstructive sleep apnea (OSA) experience recurrent episodes of apnea and hypopnea during sleep, which increase sympathetic nervous system activity and disrupt the normal neuroregulatory rhythms. This disruption may impair the function of cholinergic neurons responsible for maintaining wakefulness. Since a substantial number of cholinergic neurons are also involved in the olfactory transmission system, OSA may contribute to olfactory dysfunction through its impact on these neurons (ii) Granule cells in the olfactory bulb, functioning as interneurons, are the primary γ-aminobutyric acid (GABA)-ergic neurons that inhibit the excessive firing of mitral cells, thereby modulating the transmission of olfactory signals to higher brain centers. Additionally, in the central nervous system, recent studies have shown that GABA can activate metabotropic GABA receptors (GBB-1) in glial cells, inducing neuroprotective effects through trophic mechanisms. GABA may also mediate olfactory adaptation via ionotropic GABA receptors (LGC-38) in glial cells. In patients with obstructive sleep apnea (OSA), reduced levels of GABA have been observed in the cerebral cortex, suggesting a potential association between GABA deficits and olfactory dysfunction in OSA	Chow M et al.[26] Ahnaou, A et al.[31] Qian L et al.[32] Versace V et al.[33] Cheng H et al.[34] Macey PM et al.[35]
Disorder of Nasal Airflow in OSA Patients Leads to Olfactory Dysfunction	Deviations in the nasal septum, hypertrophy of the nasal turbinates, nasal polyps, and nasal tumors can all cause nasal obstruction, leading to alterations in nasal airflow. The minimum cross-sectional area (MCA) of the nasal cavity and nasal volume (NV) are closely linked to olfactory function. This suggests that changes in nasal airflow in patients with obstructive sleep apnea (OSA) may contribute to olfactory dysfunction	Fu D et al.[37] Wu S et al.[38] Ebihara T et al.[39]
The mutual influence of neuroinflammation and oxidative stress leads to olfactory dysfunction in patients with obstructive sleep apnea (OSA)	During the occurrence of OSA, multiple inflammatory factors are released under the influence of neuroinflammation, which can induce microglia to elevate the expression levels of pro-inflammatory cytokines. This further induces the production of interleukin-1β (IL-1β), tumor necrosis factor-alpha (TNF-α), and nitric oxide (NO). Notably, the activation of the nuclear factor kappa B (NF-κB) signaling pathway mediates the downstream release of tumor necrosis factor, interleukins, and other factors, leading to oxidative stress and synaptic failure. Conversely, due to the pivotal role of the NF-κB pathway in oxidative stress and inflammatory responses, reactive oxygen species (ROS) generated by OSA-induced oxidative stress can also act as signaling molecules, activating multiple inflammation-related signaling pathways. Under the influence of the transcription factor NF-κB, the expression of related inflammatory genes is initiated, releasing various inflammatory factors and thereby exacerbating the inflammatory response	Ahmad et al.[44] Ming, S et al.[45]
The Interaction Between Oxidative Stress and Neuroinflammation in OSA Patients Influences Neuronal Degeneration	(i)In patients with OSA, the drastic changes in oxygen tension lead to the generation of a large amount of ROS, which can directly damage the mtDNA in neuronal mitochondria. The accumulation of mtDNA mutations may result in reduced efficiency of the electron transport chain, decreased ATP production, and increased ROS generation, forming a positive feedback loop that further increases mtDNA mutations and ROS production, ultimately leading to cell death (ii)When mitochondria are damaged by ROS, their contents are released and recognized by hexokinase (HK), a glycolytic enzyme located on the outer mitochondrial membrane. This recognition further activates the NLRP3 inflammasome, triggering the release of the inflammatory cytokine IL-1β. The release of inflammatory cytokines can lead to mitochondrial dysfunction and increased ROS production, thereby forming a toxic feedback mechanism that exacerbates the loss of mitochondrial function and results in neuronal cell death	Hahn et al.[46] Mikhed Y[47] Zhou[48] He, J[49]

## References

[1] Jordan AS, McSharry DG, Malhotra A. Adult obstructive sleep apnoea. Lancet (London, England). 2014;383(9918):736–47. 10.1016/S0140-6736(13)60734-5. 23910433 10.1016/S0140-6736(13)60734-5 PMC3909558

[2] Chang K, Zaikos T, Kilner-Pontone N, Ho CY. Mechanisms of COVID-19-associated olfactory dysfunction. Neuropath Appl Neuro. 2024. 10.1111/nan.12960. 10.1111/nan.12960 PMC10906737 38419211

[3] Zuo L, Guo P, Liu L, Yu S, Lian T, Yu Q, Hu Y, Jin Z, Wang R, Piao Y, Li L, Wang Y, Wang X, Zhang W. P4–132: analyses of clinical features and investigations on potential mechanisms in patients with alzheimer’s disease and olfactory dysfunction. Alzheimers Dement. 2006. 10.1016/j.jalz.2018.06.2536. 10.3233/JAD-180425 30347619

[4] Colín-Barenque L, Pedraza-Chaverri J, Medina-Campos O, Jimenez-Martínez R, Bizarro-Nevares P, González-Villalva A, Rojas-Lemus M, Fortoul TI. Functional and morphological olfactory bulb modifications in mice after vanadium inhalation. Toxicol Pathol. 2014;43(2):282–91. 10.1177/0192623314548668. 25492423 10.1177/0192623314548668

[5] Schönenberger MJ, Kovacs WJ. Hypoxia signaling pathways: modulators of oxygen-related organelles. Front Cell Dev Biol. 2015;3:42. 10.3389/fcell.2015.00042. 26258123 10.3389/fcell.2015.00042 PMC4508581

[6] Pan Y, Lu Y, Zhou JD, Wang CX, Wang JQ, Fukunaga A, Yodoi J, Tian H. Prospect of thioredoxin as a possibly effective tool to combat OSAHS. Sleep Breath. 2022;27(2):421–9. 10.1007/s11325-022-02640-z. 35624400 10.1007/s11325-022-02640-z

[7] Singh A, Kukreti R, Saso L, Kukreti S. Oxidative stress: a key modulator in neurodegenerative diseases. Molecules. 2019. 10.3390/molecules24081583. 31013638 10.3390/molecules24081583 PMC6514564

[8] Zhao J, Yu S, Zheng Y, Yang H, Zhang J. Oxidative modification and its implications for the neurodegeneration of parkinson’s disease. Mol Neurobiol. 2016;54(2):1404–18. 10.1007/s12035-016-9743-3. 26843115 10.1007/s12035-016-9743-3

[9] Paithankar JG, Saini S, Dwivedi S, Sharma A, Chowdhuri DK. Heavy metal associated health hazards: An interplay of oxidative stress and signal transduction. Chemosphere. 2020;262: 128350. 10.1016/j.chemosphere.2020.128350. 33182141 10.1016/j.chemosphere.2020.128350

[10] Zhang H, Lei S, Zhuo H, Xu Y, Ye Y, Luo Y. TRIM24 Up-regulates ORM2 to alleviate abnormal lipid metabolism, inflammation, and oxidative stress in mice with obstructive sleep apnea syndrome and metabolic dysfunction-associated steatotic liver disease. Am J Pathol. 2024. 10.1016/j.ajpath.2024.07.020. 39168366 10.1016/j.ajpath.2024.07.020

[11] Akinsulie OC, Shahzad S, Ogunleye SC, Oladapo IP, Joshi M, Ugwu CE, Gbadegoye JO, Hassan FO, Adeleke R, Afolabi Akande Q, Adesola RO. Crosstalk between hypoxic cellular micro-environment and the immune system: a potential therapeutic target for infectious diseases. Front Immunol. 2023;14:1224102. 10.3389/fimmu.2023.1224102. 37600803 10.3389/fimmu.2023.1224102 PMC10434535

[12] Yang D, Su J, Chen Y, Chen G. The NF-κB pathway: Key players in neurocognitive functions and related disorders. Eur J Pharmacol. 2024. 10.1016/j.ejphar.2024.177038. 39369877 10.1016/j.ejphar.2024.177038

[13] Long J, Sun Y, Liu S, Yang S, Chen C, Zhang Z, Chu S, Yang Y, Pei G, Lin M, Yan Q, Yao J, Lin Y, Yi F, Meng L, Tan Y, Ai Q, Chen N. Targeting pyroptosis as a preventive and therapeutic approach for stroke. Cell Death Discov. 2023. 10.1038/s41420-023-01440-y. 37165005 10.1038/s41420-023-01440-y PMC10172388

[14] Ding X, Chen C, Zhao H, Dai B, Ye L, Song T, Huang S, Wang J, You T. Inhibiting SHP2 reduces glycolysis, promotes microglial M1 polarization, and alleviates secondary inflammation following spinal cord injury in a mouse model. Neural Regen Res. 2024;20(3):858–72. 10.4103/NRR.NRR-D-23-01925. 38886958 10.4103/NRR.NRR-D-23-01925 PMC11433905

[15] Jang SS, Pak KS, Strom A, Gomez L, Kim K, Doherty TA, DeConde AS, Ryan AF, Yan CH. Pro-inflammatory markers associated with COVID-19-related persistent olfactory dysfunction. Int Forum Allergy RH. 2023;14(4):786–93. 10.1002/alr.23264. 10.1002/alr.23264 PMC10918027 37676246

[16] Lv H, Liu P, Zhou F, Gao Z, Fan W, Xu Y. TAK-242 ameliorates olfactory dysfunction in a mouse model of allergic rhinitis by inhibiting neuroinflammation in the olfactory bulb. Int Immunopharmacol. 2021;92: 107368. 10.1016/j.intimp.2021.107368. 33454639 10.1016/j.intimp.2021.107368

[17] Sousa Garcia D, Chen M, Smith AK, Lazarini PR, Lane AP. Role of the type I tumor necrosis factor receptor in inflammation-associated olfactory dysfunction. Int Forum Allergy RH. 2016;7(2):160–8. 10.1002/alr.21855. 10.1002/alr.21855 PMC5400011 27671548

[18] Dong Y, Yu H, Li X, Bian K, Zheng Y, Dai M, Feng X, Sun Y, He Y, Yu B, Zhang H, Wu J, Yu X, Wu H, Kong W. Hyperphosphorylated tau mediates neuronal death by inducing necroptosis and inflammation in Alzheimer’s disease. J Neuroinflammation. 2022;19(1):205. 10.1186/s12974-022-02567-y. 35971179 10.1186/s12974-022-02567-y PMC9377071

[19] Yuxiong Y, Xujin X, Yi T, Ya C, Yujuan L, Shanshan H, Huiwen W. Brain-specific TRAF7 deletion ameliorates traumatic brain injury by suppressing MEKK3-regulated glial inflammation and neuronal death. Int Immunopharmacol. 2021;103: 108219. 10.1016/j.intimp.2021.108219. 34953447 10.1016/j.intimp.2021.108219

[20] Gupta KK, Khan MA, Singh SK. Constitutive inflammatory cytokine storm: a major threat to human health. J Interf Cytok Res. 2019;40(1):19–23. 10.1089/jir.2019.0085. 10.1089/jir.2019.0085 31755797

[21] Araújo B, Caridade-Silva R, Soares-Guedes C, Martins-Macedo J, Gomes ED, Monteiro S, Teixeira FG. Neuroinflammation and parkinson’s disease-from neurodegeneration to therapeutic opportunities. Cells. 2022. 10.3390/cells11182908. 36139483 10.3390/cells11182908 PMC9497016

[22] Song XY, Sun Q, Wei SZ, Wang HR, Wang Y, Zhang WB, Ren C, Song XC, Mou YK. IL-6 mediates olfactory dysfunction in a mouse model of allergic rhinitis. Brain Res. 2024;1833: 148885. 10.1016/j.brainres.2024.148885. 38531465 10.1016/j.brainres.2024.148885

[23] Soler ZM, Yoo F, Schlosser RJ, et al. Correlation of mucus inflammatory proteins and olfaction in chronic rhinosinusitis. Int Forum Allergy RH. 2020. 10.1002/alr.22499. 10.1002/alr.22499 PMC7145735 31856395

[24] Smith PA. K+ Channels in primary afferents and their role in nerve injury-induced pain. Front Cell Neurosci. 2020;14: 566418. 10.3389/fncel.2020.566418. 33093824 10.3389/fncel.2020.566418 PMC7528628

[25] Lima TS. Beyond an inflammatory mediator: Interleukin-1 in neurophysiology. Exp Physiol. 2023;108(7):917–24. 10.1113/EP090780. 37031383 10.1113/EP090780 PMC10988528

[26] Mamelak M. Sleep, narcolepsy, and sodium oxybate. Curr Neuropharmacol. 2022;20(2):272–91. 10.2174/1570159X19666210407151227. 33827411 10.2174/1570159X19666210407151227 PMC9413790

[27] Huang Y, Shen C, Zhao W, Shang Y, Wang Y, Zhang HT, Ouyang R, Liu J. Genes associated with altered brain structure and function in obstructive sleep apnea. Biomedicines. 2023. 10.3390/biomedicines12010015. 38275376 10.3390/biomedicines12010015 PMC10812994

[28] Lin C, Huang Y, Lin Q. The impact of tonsillectomy and/or adenoidectomy on cognitive function and brain structure in pediatric patients with OSAHS. Technol Health Care. 2024. 10.3233/THC-241028. 39302401 10.3233/THC-241028

[29] Satoh K, Kiyokage E, Ichikawa S, Toida K. Variations in GABA immunoreactivity among granule cells of the mouse olfactory bulb, as revealed by high-voltage electron microscopy. NEUROSCI LETT. 2020;738: 135386. 10.1016/j.neulet.2020.135386. 32947006 10.1016/j.neulet.2020.135386

[30] Harvey JD, Heinbockel T. Neuromodulation of synaptic transmission in the main olfactory bulb. Int J Environ Res Public Health. 2018. 10.3390/ijerph15102194. 30297631 10.3390/ijerph15102194 PMC6210923

[31] Ahnaou A, Chave L, Manyakov NV, Drinkenburg WHIM. Odour retrieval processing in mice: cholinergic modulation of oscillatory coupling in olfactory bulb-piriform networks. Neuropsychobiology. 2021. 10.1159/000513511. 33588406 10.1159/000513511

[32] Qian L, Rawashdeh O, Kasas L, Milne MR, Garner N, Sankorrakul K, Marks N, Dean MW, Kim PR, Sharma A, Bellingham MC, Coulson EJ. Cholinergic basal forebrain degeneration due to sleep-disordered breathing exacerbates pathology in a mouse model of Alzheimer’s disease. Nat Commun. 2022;13(1):6543. 10.1038/s41467-022-33624-y. 36323689 10.1038/s41467-022-33624-y PMC9630433

[33] Versace V, Langthaler PB, Sebastianelli L, Golaszewski S, Kunz AB, Brigo F, Saltuari L, Nardone R. Cholinergic neurotransmission and olfactory function in obstructive sleep apnea syndrome: a TMS study. SLEEP MED. 2017;37:113–8. 10.1016/j.sleep.2017.06.021. 28899520 10.1016/j.sleep.2017.06.021

[34] Cheng H, Chen D, Li X, Al-Sheikh U, Duan D, Fan Y, Zhu L, Zeng W, Hu Z, Tong X, Zhao G, Zhang Y, Zou W, Duan S, Kang L. Phasic/tonic glial GABA differentially transduce for olfactory adaptation and neuronal aging. Neuron. 2024;112(9):1473-1486.e6. 10.1016/j.neuron.2024.02.006. 38447577 10.1016/j.neuron.2024.02.006

[35] Macey PM, Sarma MK, Nagarajan R, Aysola R, Siegel JM, Harper RM, Thomas MA. Obstructive sleep apnea is associated with low GABA and high glutamate in the insular cortex. J SLEEP RES. 2016;25(4):390–4. 10.1111/jsr.12392. 26843332 10.1111/jsr.12392 PMC4974152

[36] Lim ZF, Rajendran P, Musa MY, Lee CF. Nasal airflow of patient with septal deviation and allergy rhinitis. Visual Comput Indus, Biomed, Art. 2021;4(1):14. 10.1186/s42492-021-00080-2. 10.1186/s42492-021-00080-2 PMC8137764 34014417

[37] Fu D, Pinto JM, Wang L, Chen G, Zhan X, Wei Y. The effect of nasal structure on olfactory function in patients with OSA. EUR Arch Oto-Rhino-L. 2014;272(2):357–62. 10.1007/s00405-014-3096-1. 10.1007/s00405-014-3096-1 24890976

[38] Wu S, Wang P, Xie D, Jian F. Correlation analysis of flow parameters in the olfactory cleft and olfactory function. Sci Rep. 2022;12(1):20819. 10.1038/s41598-022-25282-3. 36460767 10.1038/s41598-022-25282-3 PMC9716511

[39] Ebihara T, Omura K, Nishijima H, Yamamoto T, Otori N, Kikuta S. A new surgical technique to increase airflow in the olfactory cleft: superior turbinate lateralization procedure. EUR Arch Oto-Rhino-L. 2024. 10.1007/s00405-024-08848-x. 10.1007/s00405-024-08848-x 39017995

[40] Nishijima H, Kondo K, Yamamoto T, Nomura T, Kikuta S, Shimizu Y, Mizushima Y, Yamasoba T. Influence of the location of nasal polyps on olfactory airflow and olfaction. Int Forum Allergy RH. 2018;8(6):695–706. 10.1002/alr.22089. 10.1002/alr.22089 29394000

[41] Gillman GS, Bakeman AE, Soose RJ, Wang EW, Schaitkin BM, Lee SE, Chang YF, Mims MM. Will nasal airway surgery improve my sense of smell? A prospective observational study. Int Forum Allergy RH. 2022;13(8):1511–7. 10.1002/alr.23115. 10.1002/alr.23115 36413461

[42] Valsamidis K, Printza A, Titelis K, Constantinidis J, Triaridis S. Olfaction and quality of life in patients with nasal septal deviation treated with septoplasty. Am J Otolaryng. 2019. 10.1016/j.amjoto.2019.07.008. 10.1016/j.amjoto.2019.07.008 31345588

[43] Teleanu DM, Niculescu AG, Lungu II, et al. An overview of oxidative stress, neuroinflammation, and neurodegenerative diseases. Int J Mol Sci. 2022. 10.3390/ijms23115938. 35682615 10.3390/ijms23115938 PMC9180653

[44] Ahmad S, Choe K, Badshah H, et al. Physcion mitigates LPS-induced neuroinflammation, oxidative stress, and memory impairments via TLR-4/NF-кB signaling in adult mice. Pharmaceuticals (Basel). 2024. 10.3390/ph17091199. 10.3390/ph17091199 PMC11434929 39338361

[45] Ming S, Tian J, Ma K, et al. Oxalate-induced apoptosis through ERS-ROS-NF-κB signalling pathway in renal tubular epithelial cell. Mol Med. 2022. 10.1186/s10020-022-00494-5. 35922749 10.1186/s10020-022-00494-5 PMC9347104

[46] Hahn A, Zuryn S. Mitochondrial genome (mtDNA) mutations that generate reactive oxygen species. Antioxidants (Basel). 2019. 10.3390/antiox8090392. 31514455 10.3390/antiox8090392 PMC6769445

[47] Mikhed Y, Daiber A, Steven S. Mitochondrial oxidative stress, mitochondrial DNA damage and their role in age-related vascular dysfunction. Int J Mol Sci. 2015. 10.3390/ijms160715918. 26184181 10.3390/ijms160715918 PMC4519931

[48] Zhou R, Yazdi AS, Menu P, et al. A role for mitochondria in NLRP3 inflammasome activation. Nature. 2010;469(7329):221–5. 10.1038/nature09663. 21124315 10.1038/nature09663

[49] He J, Zhu G, Wang G, et al. Oxidative stress and neuroinflammation potentiate each other to promote progression of dopamine neurodegeneration. OXID MED CELL LONGEV. 2020. 10.1155/2020/6137521. 32714488 10.1155/2020/6137521 PMC7354668

[50] McWhorter FY, Davis CT, Liu WF. Physical and mechanical regulation of macrophage phenotype and function. CMLS-CELL MOL LIFE S. 2014;72(7):1303–16. 10.1007/s00018-014-1796-8. 10.1007/s00018-014-1796-8 PMC4795453 25504084

[51] Ho MS. Microglia in parkinson’s disease. Adv Exp Med Biol. 2019;1175:335–53. 10.1007/978-981-13-9913-8_13. 31583594 10.1007/978-981-13-9913-8_13

[52] Subramaniam SR, Federoff HJ. Targeting microglial activation states as a therapeutic avenue in parkinson’s disease. Front Aging Neurosci. 2017. 10.3389/fnagi.2017.00176. 28642697 10.3389/fnagi.2017.00176 PMC5463358

[53] Block ML, Hong JS. Chronic microglial activation and progressive dopaminergic neurotoxicity. Biochem Soc T. 2007;35(Pt 5):1127–32. 10.1042/BST0351127. 10.1042/BST0351127 17956294

[54] Yang J, Han Y, Diao X, et al. Screening of obstructive sleep apnea and diabetes mellitus -related biomarkers based on integrated bioinformatics analysis and machine learning. Sleep Breath. 2025. 10.1007/s11325-024-03240-9. 39804507 10.1007/s11325-024-03240-9 PMC11729194

[55] Boppana TK, Mittal S, Madan K, et al. Antioxidant therapies for obstructive sleep apnea: A systematic review and meta-analysis. Sleep Breath. 2024. 10.1007/s11325-024-03050-z. 38740632 10.1007/s11325-024-03050-z

[56] Wei RM, Zhang MY, Fang SK, et al. Melatonin attenuates intermittent hypoxia-induced cognitive impairment in aged mice: The role of inflammation and synaptic plasticity. Psychoneuroendocrino. 2025. 10.1016/j.psyneuen.2024.107210. 10.1016/j.psyneuen.2024.107210 39378690

[57] Ahmad S, Choe K, Badshah H, Ahmad R, Ali W, Rehman IU, Park TJ, Park JS, Kim MO. Physcion mitigates LPS-induced neuroinflammation, oxidative stress, and memory impairments via TLR-4/NF-кB signaling in adult mice. Pharmaceuticals (Basel). 2024. 10.3390/ph17091199. 10.3390/ph17091199 PMC11434929 39338361

